# Adaptation to chronic acidic extracellular pH elicits a sustained increase in lung cancer cell invasion and metastasis

**DOI:** 10.1007/s10585-019-09990-1

**Published:** 2019-09-05

**Authors:** Shusaku Sutoo, Toyonobu Maeda, Atsuko Suzuki, Yasumasa Kato

**Affiliations:** 1 Department of Oral Function and Molecular Biology, Ohu University School of Dentistry, Koriyama, 963-8611 Japan; 2 Department of Oral Physiology and Biochemistry, Ohu University Graduate School of Dentistry, Koriyama, 963-8611 Japan; 3 Department of Oral Rehabilitation, Ohu University Graduate School of Dentistry, Koriyama, 963-8611 Japan

**Keywords:** MMP-9, Acidic extracellular pH, Adaptation, LLC, Metastasis

## Abstract

Acidic extracellular pH (pH_*e*_) is an important microenvironment for cancer cells. This study assessed whether adaptation to acidic pH_*e*_ enhances the metastatic phenotype of tumor cells. The low metastatic variant of Lewis lung carcinoma (LLCm1) cells were subjected to stepwise acidification, establishing acidic pH_*e*_-adapted (LLCm1A) cells growing exponentially at pH 6.2. These LLCm1A cells showed increased production of matrix metalloproteinases (MMPs), including MMP-2, -3, -9, and -13, and pulmonary metastasis following injection into mouse tail veins. Although LLCm1A cells exhibited a fibroblastic shape, keratin-5 expression was increased and α-smooth muscle actin expression was reduced. Despite serial passage of these cells at pH 7.4, high invasive activity through Matrigel^®^ was sustained for at least 28 generations. Thus, adaptation to acidic pH_*e*_ resulted in a more invasive phenotype, which was sustained during passage at pH 7.4, suggesting that an acidic microenvironment at the primary tumor site is important in the acquisition of a metastatic phenotype.

**Electronic supplementary material:**

The online version of this article (10.1007/s10585-019-09990-1) contains supplementary material, which is available to authorized users.

## Introduction

Extracellular pH (pH_*e*_) becomes acidic due to excess cellular glycolysis. In the presence of oxygen, lactic acid is the main cause of extracellular acidification, a process called the “Warburg effect” or “aerobic glycolysis” [1]. Because the expression of most glycolytic enzymes is driven by hypoxia inducible factor-1 (HIF-1), extracellular acidification is closely related to hypoxia [1]. Among lactate anion/H^+^ symporters, also known as monocarboxylate transporters (MCTs), the hypoxia-inducible subtype MCT4 is primarily responsible for the secretion of lactic acid. MCT4 exports lactate, thereby affecting the proliferation of tumor cells [2]. An alternative major cause of extracellular acidity in tumor tissue results from the hydration of CO_2_ by tumor carbonic anhydrase IX [3, 4]. HIF-1 activation in tumors up-regulates angiogenesis and/or lymphangiogenesis. These newly formed vessels provide primary tumor cells the opportunity to disseminate through the circulation [5]. Acidic pH_*e*_ also induces the production of vascular endothelial cell growth factor (VEGF)-A [6], interleukin-8 (IL-8) [7], and VEGF-C [8] through an HIF-1 independent pathway. Thus, an acidic pH_*e*_ microenvironment, whether independent of, in addition to, or synergistically with hypoxia, may support the malignant phenotype of cancer cells and play a role in metastasis.

Tumor-derived acidic pH_*e*_ can act as a feed-back stimulator of a metastatic phenotype. Our investigations of the association of acidic pH_*e*_ with the metastasis-related activities of mouse B16 melanoma variants, including the induction of matrix metalloproteinase-9 (MMP-9) expression, found that MMP-9 induction correlated with the metastatic activity of B16 variants and the acceleration of tumor invasion through type IV collagen sheets [9, 10]. Transient exposure to acidic pH_*e*_ resulted in a switch from an epithelial to a mesenchymal phenotype, called an epithelial-mesenchymal transition (EMT) [11–13]. Transient acidic pH_e_ 5.9–6.8 was found to potentiate the invasive and metastatic activities of these cells [8, 12, 14–19]. In vivo mapping of pH_e_ in mouse B16-F10 melanoma xenografts with CEST-MRI [20] showed that the pH_*e*_ of most early stage tumors ranged between pH 6.0–6.2, whereas the pH_*e*_ of most late stages tumors ranged between pH 5.7–6.7, with 10% of the area of late stage tumors having a pH_*e*_ < 5.5. These findings suggested that primary tumors were continuously influenced by pH_*e*_ 6.0–6.2 over a long period and that adaptation of tumor cells to this pH_*e*_ range is an important step in tumor metastasis.

Because an acidic microenvironment can chronically affect tumor cells in vivo, studies are needed to evaluate the chronic effects of pH_*e*_. Tumor cell lines have been subjected to chronic extracellular acidification and/or adaptation to pH_*e*_ 6.7 for 2 weeks to 3 months [21–23]. We found that the growth rates of cells were equal at pH 6.8 and pH 7.4 and that these cells could grow at pH 6.5 after recovering from a transient decrease in proliferation rate. In vivo imaging showed that pH_*e*_ 6.2 could be attained [20]. In this study, we established cells proliferating exponentially at pH 6.2 and investigated whether adaptation to acidic pH_*e*_ increased tumor metastatic activity and whether the metastatic phenotype could be sustained at neutral pH_*e*_.

## Materials and methods

### Reagents

Dulbecco’s modified Eagle’s medium (DMEM), Ham’s F12 medium, and High Capacity RNA-to-cDNA kits were purchased from Thermo Fisher Scientific (Waltham, MA, USA). SYBR Premix Ex *Taq* II was from Takara Bio (Tokyo, Japan), fetal bovine serum (FBS) was from Hyclone (South Logan, UT, USA), and sodium pentobarbital was from Kyoritsu (Tokyo, Japan).

### Cells and cell culture

A low metastatic variant of Lewis lung carcinoma (LLCm1) was established in our laboratory using an experimental lung metastasis method through tail vein injection [12]. Basal medium was prepared as described. Briefly, a 1:1 mixture of DMEM and F12 was supplemented with 15 mM HEPES, 4 mM H_3_PO_4_ 1.0 g/L NaHCO_3_, 100 units/mL penicillin G, and 0.1 mg/mL streptomycin sulfate, and its pH was adjusted with NaOH or HCl [14]. Cells were serially passaged with 0.05% trypsin/0.02% EDTA and cultured in the presence of 10% FBS at 37 °C in a humidified atmosphere in a 5% CO_2_ incubator.

Cells were adapted to acidic pH_*e*_ by serial passage through media of stepwise decreasing pH (7.0, 6.8, and 6.5) until pH 6.2 was reached. The cells were maintained for 2–4 weeks at each pH and passaged 2–3 times per week, depending on growth rate. Adaptation to each pH_*e*_ was confirmed by showing exponential growth after seeding cells at 2.5 × 10^5^ cells/60 mm dish. Finally, acidic pH_*e*_-adapted cells (LLCm1A cells) were established by more than 40 passages (more than 3 months) through medium at pH 6.2 in the presence of 10% FBS. Where indicated, LLCm1A cells were passaged 3–10 times in medium at pH 7.4 in the presence of 10% FBS.

### Growth curve and doubling time

Cells were suspended in medium at pH 7.4 containing 10% FBS and seeded onto 24-well plates. After 3 h, the medium was changed to medium of various pH containing 10% FBS. At this time, cells in some wells were counted and determined as the cell number at day 0. Cells were harvested using trypsin/EDTA and the number of cells in each well counted using the trypan blue dye exclusion method. Doubling time was calculated as (T_*1*_ − T_*0*_)/log2 (N_*1*_/N_*0*_), with N_*0*_ and N_*1*_ defined as the number of cells at the initial time (T_*0*_) and after cultivation for time T (T_*1*_), respectively.

### Lung metastasis

All animal experiments were performed in accordance with the guidelines of the Ministry of Education, Culture, Sports, Science and Technology, the Ministry of Health, Labor and Welfare of Japan and ARRIVE [24]. The experimental protocols were approved by the Animal Experimental Committee of Ohu University (Koriyama, Japan) (#2014–15). LLCm1 and LLCm1A cells were harvested with trypsin/EDTA, resuspended in DMEM/F12 (pH 7.4) containing 10% FBS, and incubated at 37 °C for 1 h. The cells were washed twice with Mg^2+^ and Ca^2+^-free phosphate-buffered saline (PBS(-)) and resuspended in ice cold PBS(-). In experimental metastasis assays [12, 25, 26], 3 × 10^5^ cells in 200 µl PBS(-) were injected into the tail vein of each 7-week-old male C57BL/6 mouse (Clea Japan, Tokyo, Japan). Each experimental group consisting of 6 mice was housed in a cage. Animals were maintained in the barrier facility for laboratory animals with a 12 h light–dark cycle and allowed food and water ad libitum. Three weeks later, the mice were sacrificed by intraperitoneal injection of sodium pentobarbital (120 mg/kg). Their lungs were removed and the numbers of metastatic foci at lung surfaces were counted [26].

### Reverse transcription-quantitative polymerase chain reaction (RT-qPCR)

Total RNA was purified using the acid-guanidinium-thiocyanate-phenol-chloroform (AGPC) method and reverse-transcribed to cDNA using a High-Capacity cDNA Reverse Transcription Kit. Target sequences were amplified by SYBR Premix Ex *Taq* II in a Thermal Cycler Dice Real Time System (TP-870, Takara Bio) using the specific primers listed in Table S1. The level of expression of each target gene was normalized relative to the level of *Actb* mRNA in the same samples. The data were analyzed by the $2^{-\Delta C_{\text{t}}}$ method [27], with normalized expression calculated as individual data point according to the formula:

$$\Delta C_{\text{t}} = C_{{{\text{t}}\,{\text{gene}}\,{\text{of}}\,{\text{interest}}}} {-}C_{{{\text{t}}\, Actb\, {\text{gene}}}}$$

Fold gene induction = $2^{-\Delta C_{\text{t}}}$ value (Experimental group)/$2^{-\Delta C_{\text{t}}}$ value (Control group). Control group: LLCm1 cells at pH 7.4. Experimental group: LLCm1 cells at pH 6.8, LLCm1A cells at pH 7.4, or LLCm1A cells at pH 6.8

### Zymography

MMP-2 and -9 activities were determined by gelatin-zymography, as described [9, 10, 12, 26]. Briefly, cells were cultured in serum-free medium for 24 h. The proteins in the conditioned medium (CM) were concentrated by acetone precipitation and separated by electrophoresis in gelatin-containing 7.5% polyacrylamide-sodium dodecyl sulfate (SDS) gels, without prior heating or reduction. Loading quantity was adjusted to cell density in each experiment. After electrophoresis, the gels were washed with 2.5% Triton-X100 in Tris–HCl (pH 7.5), 5 mM NaCl to remove SDS, incubated in 50 mM Tris–HCl (pH 7.5), 10 mM CaCl_2_ for 24 h at 37 °C, and stained with Coomassie Brilliant Blue R-250.

### Wound healing (scratch) assay

Wound healing assays were performed as described [12]. Briefly, confluent cultures in 6-well plates were serum-starved for 24 h and scratched with a micropipette tip. After removal of debris, the cells were cultured in medium containing 0.2% FBS at pH 7.4 or pH 6.8. Photographs were taken at 18 h and the distance between the original edge of the wound and the front line formed by cells that had migrated was measured.

### In vitro invasion assay

In vitro invasive activity was determined using Matrigel^®^-coated polycarbonate porous filters (8 μm pores) mounted onto transwell chambers (Corning, Tewksbury, MA, USA) as described [12]. Briefly, cells were serum-starved overnight at pH 7.4 and maintained in serum-free media at pH 7.4 or pH 6.8 for 18 h. The culture medium was centrifuged, and the cell suspensions were stored at 37 °C. Adherent cells were harvested with trypsin/EDTA, incubated at 37 °C for 30 min in medium containing 10% FBS, washed twice with warmed PBS(–), re-suspended in the culture medium stored at 37 °C, and inoculated at a density of 5 × 10^5^ cells/100 μl/chamber on an insert consisting of a Matrigel^®^ (37.9 μg/cm^2^)-coated filter. This insert had been mounted onto a well of a 24-well plate, which had been filled with 600 μl of 20% FBS-containing medium adjusted to the same pH as the chemoattractant. After incubation for 18 h, non-invasive cells were removed with a cotton swab and the invasive cells were fixed in 100% methanol, stained with Giemsa solution, and counted under a light microscope (× 200).

### Statistical analysis

Results were expressed as mean ± SE. Two independent samples were compared by Student’s *t*-tests, and more than two samples compared by ANOVA and the Holm method [28]. Data of in vitro assays were representative of two or more independent experiments, each of which contained triplicate samples (unless otherwise noted). P values less than 0.05 were considered statistically significant.

## Results

### Acidic pH_*e*_-adapted LLCm1 cells showed a fibroblastic morphology and increased metastatic activity

**Fig. 1:**
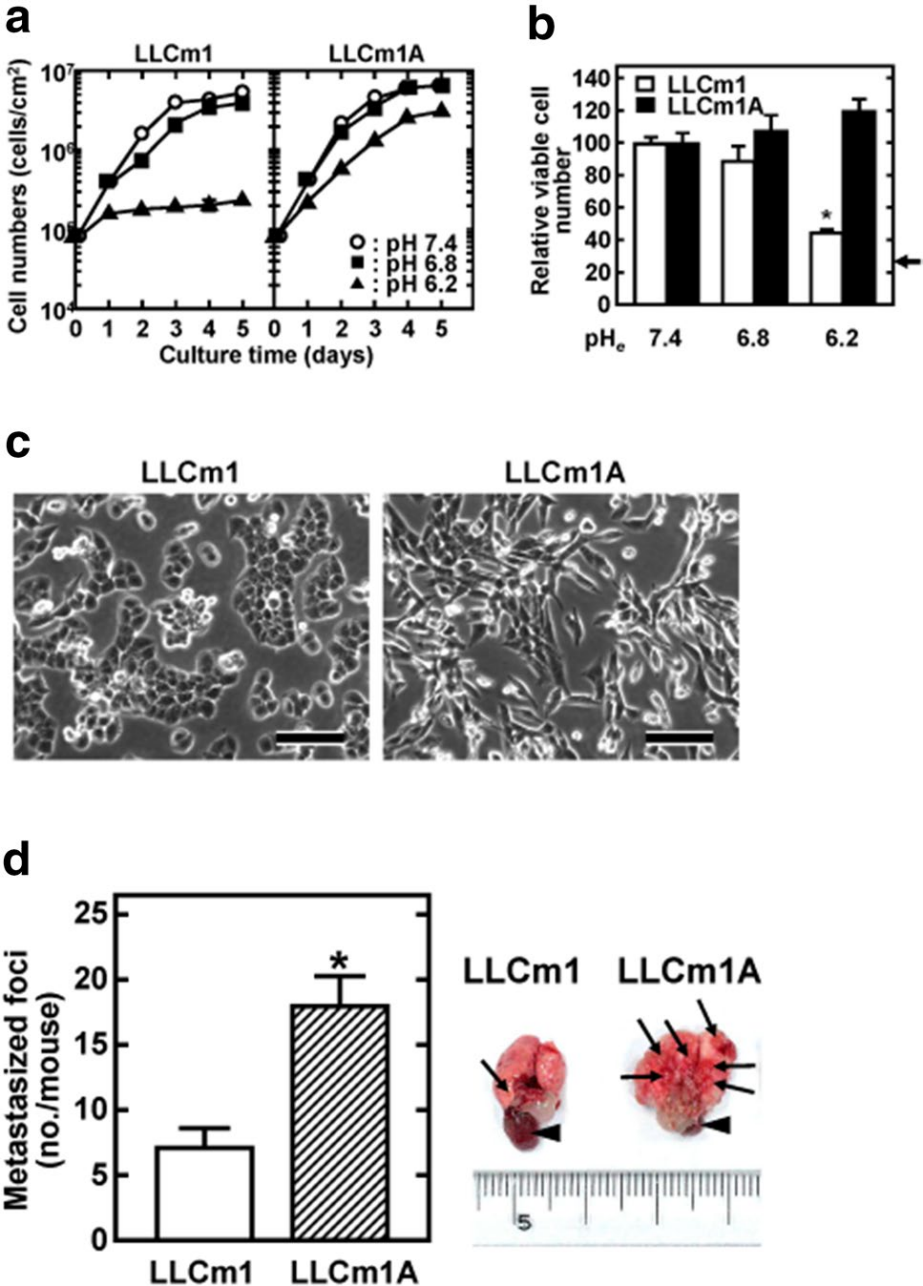
LLCm1A cells exhibit high proliferation at pH 6.2 and have a fibroblastic cell shape and increased metastatic ability. Growth curve. **a** Cells in pH 7.4 medium containing 10% FBS were seeded at 8.5 × 10^4^ cells/cm^2^ in 24-well plates. Three hours later, the culture medium was changed to pH 7.4 (control), pH 6.8 or pH 6.2 containing 10% FBS, with the media changed every day. Viable cell numbers were determined using the trypan blue dye exclusion method. **b** Cells were plated at 2.0 × 10^4^ cells/cm^2^ in 24 well plates in pH 7.4 medium containing 10% FBS. After 24 h, the culture medium was changed to pH 7.4 (control), pH 6.8 or pH 6.2 medium containing 10% FBS and the cells maintained for 24 h. Viable cell numbers were determined using the trypan blue dye exclusion method. The arrow shows the number of cells as time zero. Representative results of two independent experiments are reported as mean ± SE (n = 3). **c** Morphology. LLCm1 and LLCm1A cells were plated onto plastic dishes and cultured in pH 7.4 medium containing 10% FBS for 2 days. Phase contrast micrographs were taken. Bar, 100 μm. **d** Metastasis. LLCm1A cells were passaged for 2 weeks in pH 7.4 medium containing 10% FBS. LLCm1 and LLCm1A cells in logarithmic growth phase at pH 7.4 were harvested and 3 × 10^5^ cells were injected into the tail vein of each of six C57BL/6 mice. Three weeks later, the mice were sacrificed and the metastasized foci (shown as arrows) at the lung surfaces were counted. Arrow heads show the heart. In some cases, error bars are hidden by the data symbol due to small values. Representative results of two independent experiments are reported as mean ± SE (n = 6). *P < 0.05

To establish acidic pH_*e*_-adapted, or LLCm1A, cells, LLCm1 cells were conditioned by stepwise reductions in pH_*e*_, with the recovery of proliferative capacity confirmed at each pH_*e*_. Although LLCm1 cells continuously grew at pH_*e*_ 6.5, they were unable to grow at pH_*e*_ 6.2. A critical point was observed between pH_*e*_ 6.5 and pH_*e*_ 6.2. These cells were maintained at pH_*e*_ 6.2 by medium renewal alone until significant growth was observed. Overall, more than 3 months were required to obtain proliferating LLCm1A cells at pH_*e*_ 6.2. Acclimation involved the seeding of LLCm1 cells onto 24-well culture plates at pH_*e*_ 7.4, followed 3 h later by replacement with medium at different pH; thereafter culture media were renewed every day. An obvious reduction in growth rate was not seen until pH_*e*_ 6.5. However, cells showed almost no growth in medium at pH_*e*_ 6.2. (Figure 1a, Table 1). If, however, cells were seeded at pH 7.4, the medium changed to a different pH after 1 day and this medium renewed every other day, the cells grew, even at pH 6.2, on day 2 (the first day of acidification) but the number of viable cells was reduced on day 3 (the second day of acidification) (Fig. S1).

**Table 1:** Doubling time (h)

Day	LLCm1			LLCm1A		
	pH 7.4	pH 6.8	pH 6.2	pH 7.4	pH 6.8	pH 6.2
0–1	9.9	10.1	20.3	10.5	9.7	15.5
1–2	12.0	24.0	136.6	9.7	12.3	17.5
2–3	17.8	15.4	287.2	22.3	23.2	20.3

In contrast to parental LLCm1 cells, LLCm1A cells grew exponentially at pH_*e*_ 6.8 and at pH_*e*_ 6.2, although the doubling time at pH_*e*_ 6.2 was slower (Fig. 1a, Table 1). Lag time was not obvious when LLCm1A cells were seeded at pH_*e*_ 6.2 (Fig. S1), showing that these cells had high seeding efficiency. LLCm1A cells had a fibroblastic shape and cell-to-cell contact was dispersed. In contrast, parental LLCm1 cells showed a cobblestone like morphology (Fig. 1b). Injection of LLCm1A cells subjected to 3 passages at pH_*e*_ 7.4 into mouse tail veins gave rise to a greater number of lung metastases than parental LLCm1 cells (Fig. 1c).

### High production of matrix metalloproteinases

**Fig. 2:**
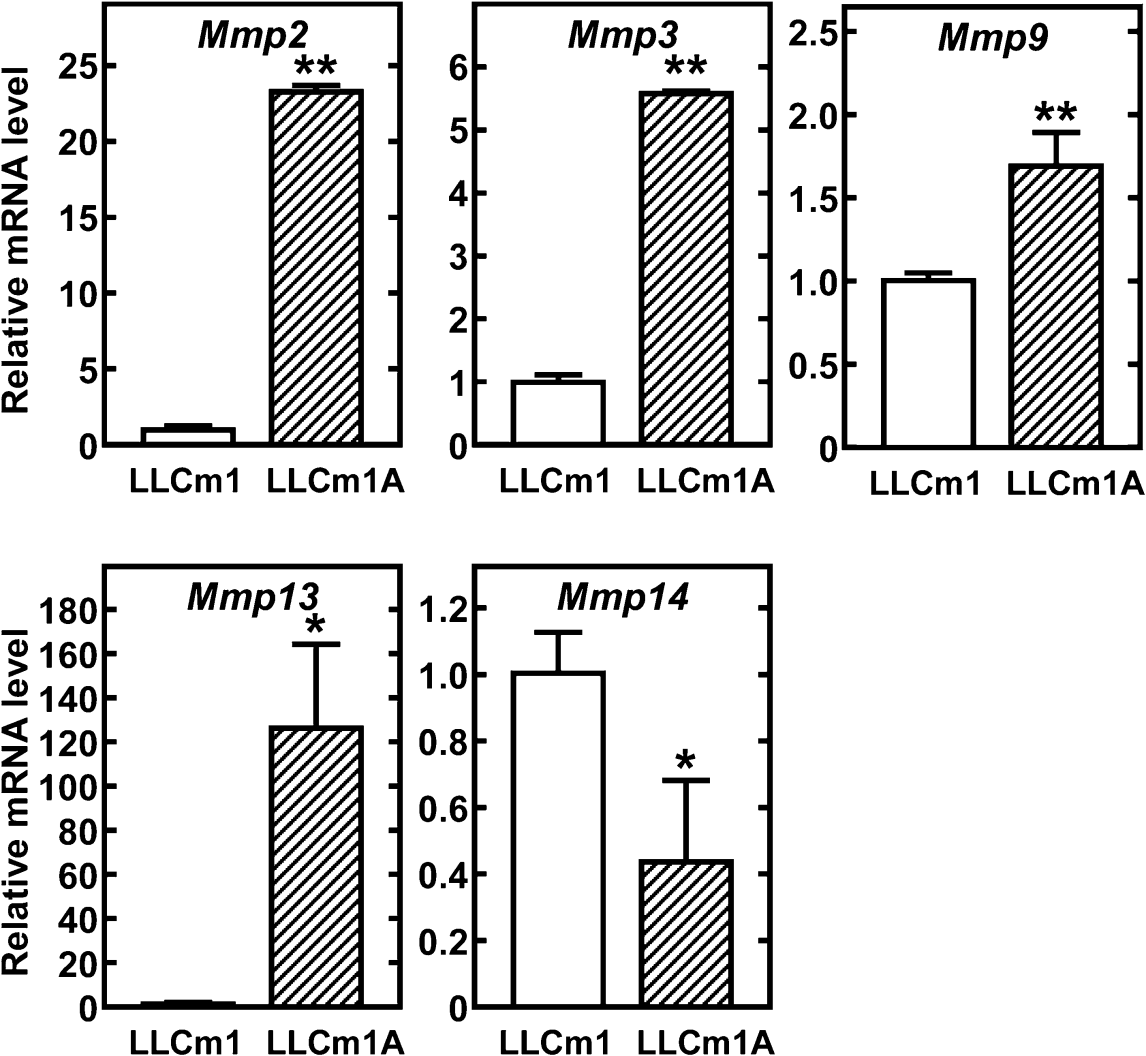
Expression of MMP mRNAs. Total RNA was purified from serum-free cultures incubated for 18 h at pH 7.4, reverse-transcribed and amplified by qPCR with specific primer sets for MMPs. Representative results of three independent experiments are reported as mean ± SE (n = 3). *P < 0.05, **P < 0.01

The expression of MMPs was compared in LLCm1A and LLCm1 cells. To avoid differences in experimental conditions, both cell types were cultured at pH_*e*_ 7.4. Expression of mRNAs encoding MMP-2, -3,-9, and -13 was higher in LLCm1A than in LLCm1 cells, whereas the level of *Mmp14* mRNA, encoding membrane type 1 (MT1)-MMP, was lower in LLCm1A than in LLCm1 cells (Fig. 2).

### Adaptation to acidic pH_*e*_ induces mesenchymal cell morphology and phenotype without typical mesenchymal marker expression

**Fig. 3:**
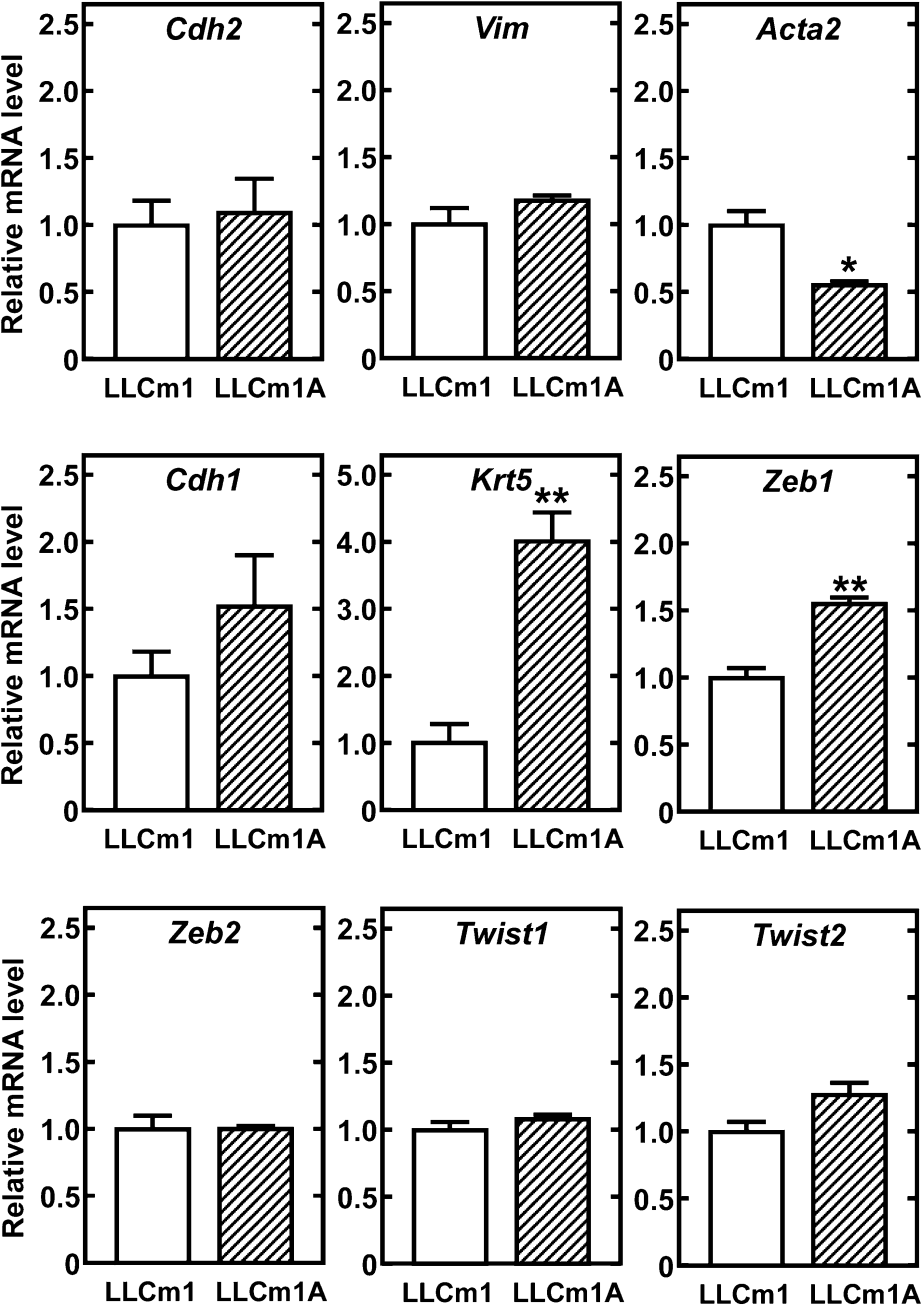
Expression of mesenchymal and epithelial marker mRNAs. Total RNA was purified from serum-free cultures incubated for 18 h at pH 7.4, reverse-transcribed and amplified by qPCR with specific primer sets for the mesenchymal markers N-cadherin (*Cdh2*), vimentin (*Vim*), and α-smooth muscle actin (*Acta2*); and the epithelial markers E-cadherin (*Pdh1*) and keratin5 (*Krt5*). Representative results of two independent experiments are reported as mean ± SE (n = 3). *P < 0.05, **P < 0.01

Because LLCm1A cells had a spindle shape with little cell-to-cell contact, their expression of mesenchymal and epithelial cell markers was investigated. Unexpectedly, the expression of *Acta2* mRNA, encoding the mesenchymal marker αSMA, was lower and the expression of *Krt5* mRNA, encoding the epithelial marker keratin-5, was higher in LLCm1A than in LLCm1 cells (Fig. 3). Although we observed a slight increase in the level of *Zeb1* mRNA, the product of which reduces the expression of *Cdh1* mRNA, encoding E-cadherin, *Cdh1* mRNA expression was not elevated. The expression of other marker mRNAs did not differ in LLCm1 and LLCm1A cells. These findings suggest that mesenchymal-epithelial transition (MET)-like changes, rather than EMT, occurred partly by adaptation to acidic pH_*e*_.

### Transient acidification further increases expression of MMPs

**Fig. 4:**
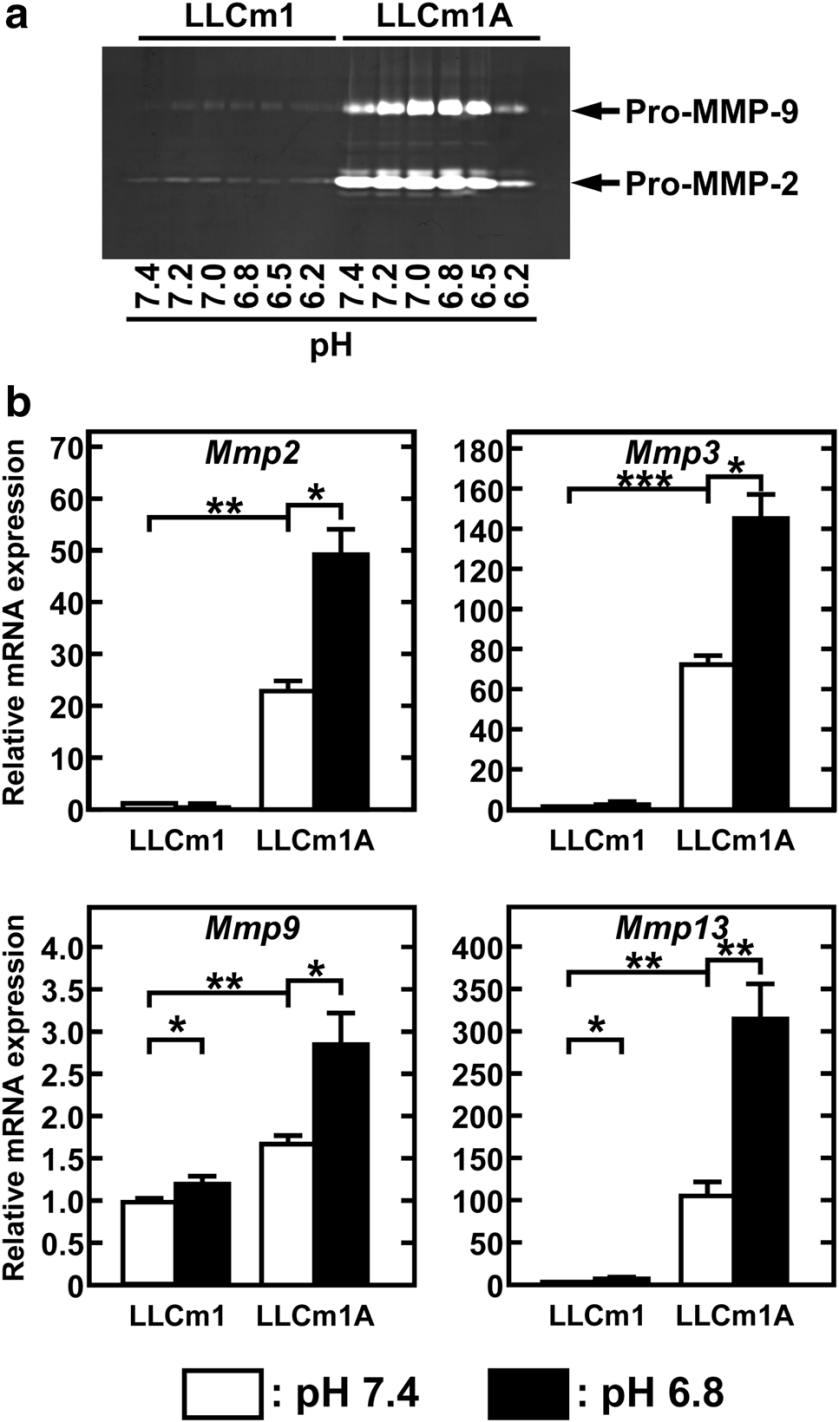
Acidic pH_*e*_ enhances expression of MMP mRNAs in LLCm1A cells. Cells pre-incubated in serum-free medium at pH 7.4 for 18 h were incubated in serum-free medium at the indicated pHs for an additional 24 h. **a** MMPs in the CM were collected, concentrated by acetone precipitation, and analyzed by gelatin-zymography. **b** Expression of mRNAs encoding MMPs was analyzed by RT-qPCR. Representative results of three independent experiments are reported as mean ± SE (n = 3). *P < 0.05, **P < 0.01

Zymographic analysis of the pH_*e*_ dependent secretion of MMP-2 and -9 showed that the production of both enzymes was highly enhanced at pH_*e*_ 6.8 (Fig. 4a). In agreement with zymographic analysis, the expression of *Mmp2* and *Mmp9* mRNAs was significantly higher in LLCm1A than in LLCm1 cells (Fig. 4b). In addition, transient acidification induced *Mmp3* and *Mmp13* mRNA expression.

### Different effects of adaptation to and transient stimulation by acidic pH_*e*_

**Fig. 5:**
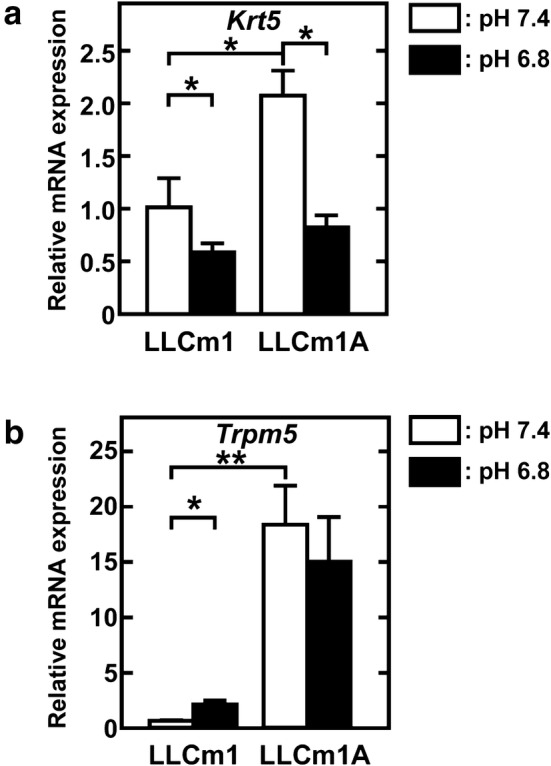
Adaptation to acidic pH_*e*_ and transient exposure to acidic pH_*e*_ have different effects. Total RNA was purified from serum-free cultures for 24 h at pH 7.4 or pH 6.8 after pre-incubation for 18 h at pH 7.4, reverse-transcribed and amplified by qPCR with specific primer sets for *Krt5* and *Trpm5*. Representative results of two independent experiments are reported as mean ± SE (n = 3). *P < 0.05, **P < 0.01

In contrast to the effects of transient acidification on MMP expression, acidification enhanced *Krt5* mRNA expression in LLCm1A cells but reduced its expression in LLCm1 cells (Fig. 5a). We recently showed that TRPM5 is important for acidic pH_*e*_ signaling and that high TRPM5 mRNA expression was associated with shorter survival of patients with some types of tumor [26]. Here, we investigated whether adaptation to acidic pH_*e*_ increased *Trpm5* mRNA expression, finding that the level of *Trpm5* mRNA expression in LLCm1A cells was not affected by transient exposure to extracellular acidification (Fig. 5b). Although LLCm1 cells responded to transient acidification with an increase in *Trpm5* mRNA, this level was only ≈ 15% of that in LLCm1A cells.

Although LLCm1 cells responded to transient acidification with an increase in *Trpm5* mRNA.

### LLCm1A cells show increased migration and in vitro invasion

**Fig. 6:**
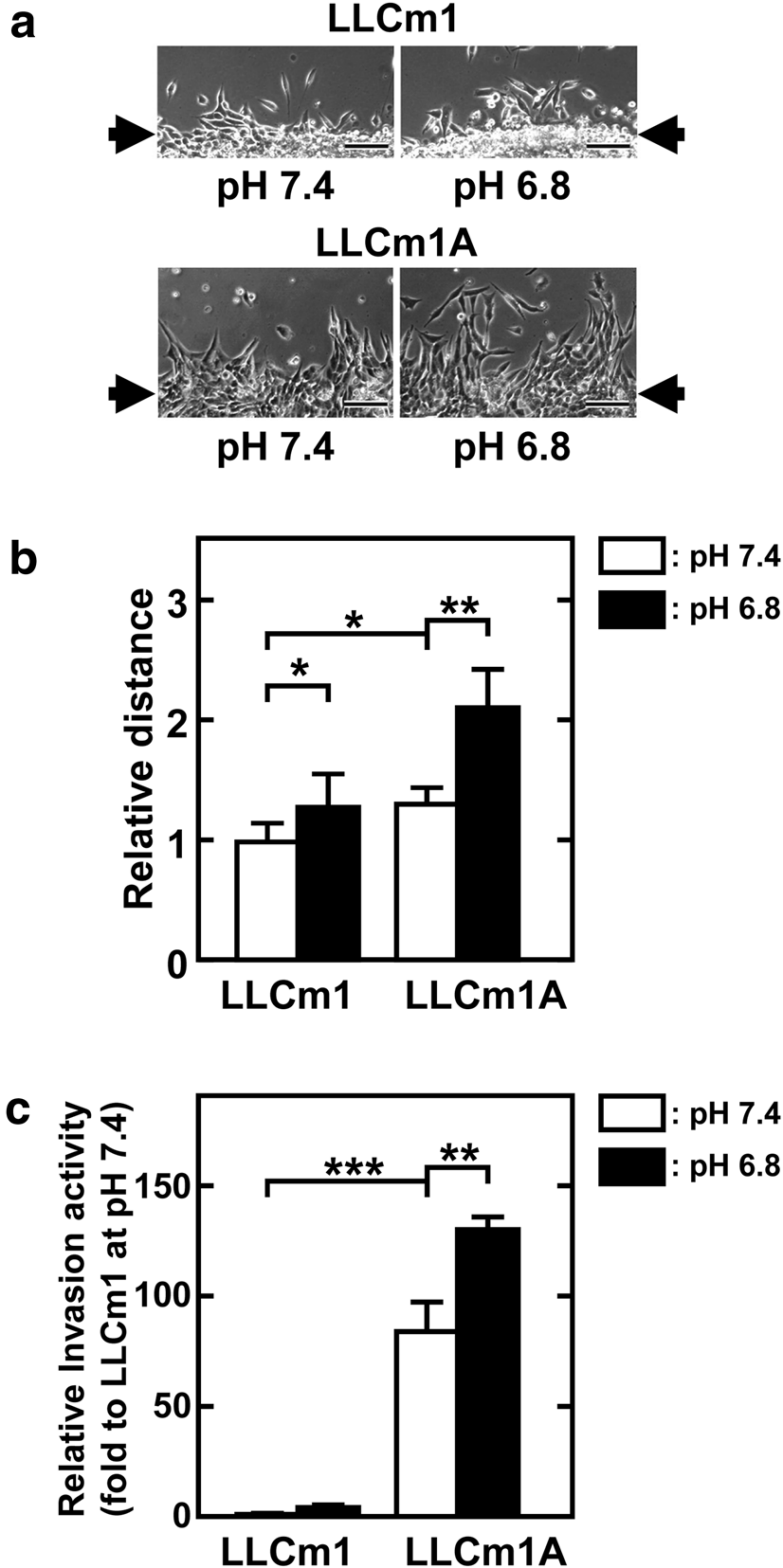
Migration and invasive activities are higher in LLCm1A than in LLCm1 cells, with these activities further increased by acidic pH_*e*_. Confluent cultures were scratched with micropipette tips and incubated for 18 h in medium at pH 7.4 or pH 6.8 containing 2% FBS. **a** Phase-contrast micrographs. **b** Relative migration distance relative to LLCm1 cells at pH 7.4 (n = 8). **c***In vitro* invasion activity through Matrigel^®^. Serum-starved cells were maintained in serum-free medium at pH 7.4 or 6.8. Medium was collected, and cells were harvested by trypsinization and suspended in the same own medium. Cells (5 × 10^5^) were placed onto Matrigel^®^-coated filters in transwell chambers. The chemoattractant was 20% FBS. Cells that passed through onto the lower surface of the filter were counted after Giemsa staining. In some cases, error bars are hidden by the data symbol due to small values. Representative results of two independent experiments are reported as mean ± SE (n = 3). *P < 0.05, **P < 0.01

**Fig. 7:**
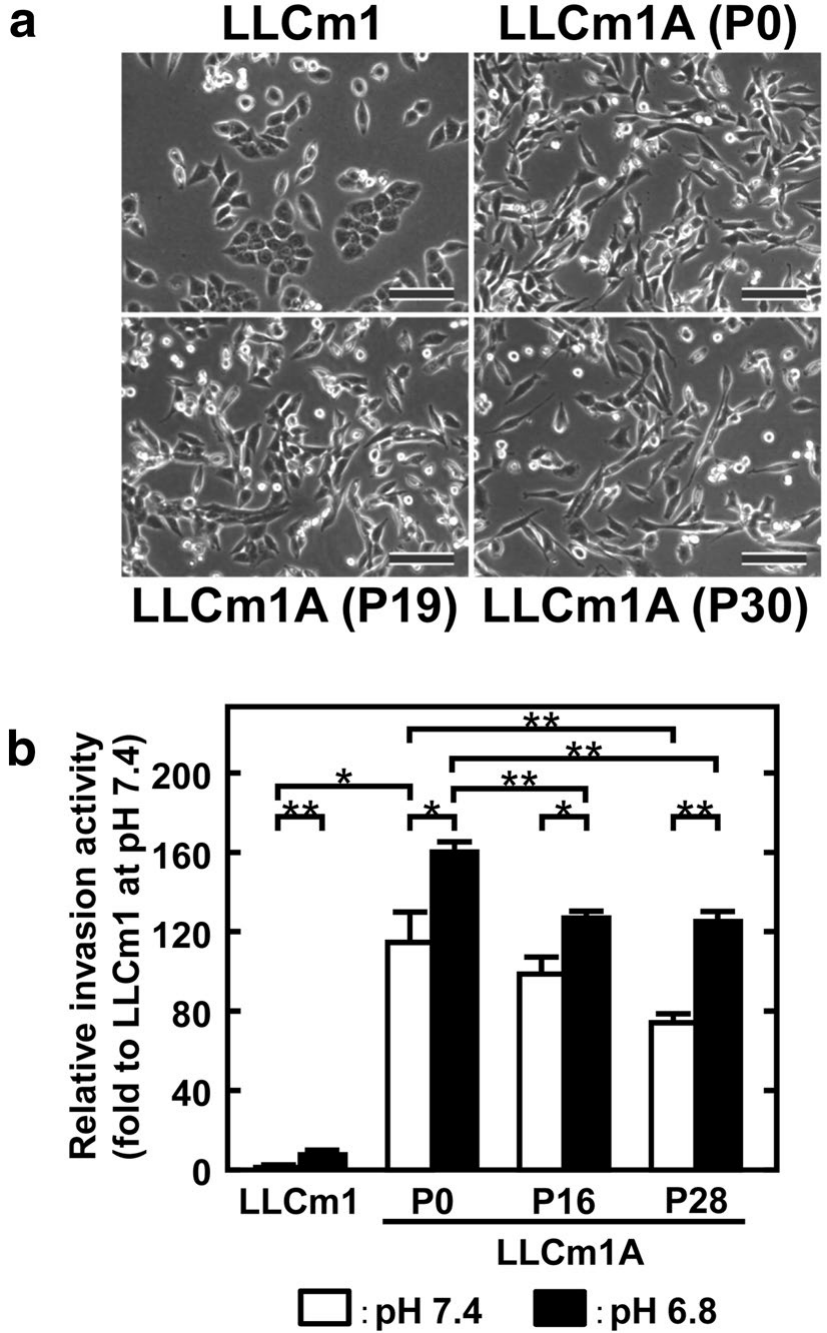
Fibroblastic morphology and high invasive activity of LLCm1A cells are sustained until late passage generation at pH 7.4. **a** Cells were plated onto plastic dishes and cultured in pH 7.4 medium containing 10% FBS for 2 days. Phase-contrast micrographs were taken. The number followed by P in round brackets indicates the number of cell passages. Bar, 100 μm. **b** Serum-starved cells were maintained in serum-free medium at pH 7.4 or 6.8. Culture medium was collected, and the cells were harvested by trypsinization and suspended in the same culture medium. Cells (5 × 10^5^) were placed onto Matrigel^®^-coated filters in transwell chambers. Cells that passed through onto the lower surface of the filter were counted after Giemsa staining. The number followed by P indicates the number of cell passages. In some cases, error bars are hidden by the data symbol due to small values. Representative results of two independent experiments are reported as mean ± SE (n = 3). *P < 0.05, **P < 0.01

We previously showed that extracellular acidification of LLCm1 cells increased their migration and invasive activities [12]. We therefore tested the migration and Matrigel^®^ invasion activities of LLCm1A cells. Scratch assays clearly showed that LLCm1A cells had greater migratory activity than LLCm1 cells (Fig. 6a, b). The activity of both cells was also upregulated by transient treatment with acidic pH_*e*_. In addition, LLCm1A cells showed higher in vitro invasive activity through Matrigel^®^ than parental LLCm1 cells (Fig. 6c), with fibroblastic morphology and invasive activity sustained after long-term passage at neutral pH_*e*_ (Fig. 7).

Because our study was designed to assess whether tumor cells exposed to acidic pH_*e*_ have increased their metastatic phenotype even at physiological pH_*e*_, such as in blood, facilitating the formation of secondary tumors, LLCm1A cells were cultured in medium containing 10% serum at pH_*e*_ 7.4 and the effects of this “switch to neutral pH_*e*_” on invasive phenotype was assessed. Unexpectedly, pH_*e*_ 6.2-adapted LLCm1A cells detached within several hours and were no longer maintained in serum-free or serum-reduced (2% FBS) conditions (Fig. 2S). In contrast, these cells spread well and could be maintained in serum-free and serum-reduced (2% FBS) conditions at pH_*e*_ 6.5. MMP-2 and -9 levels and invasive activity were high under acidic conditions (pH_*e*_ 6.5–6.8) without switching to neutral pH (Fig. 2S). Although MMP activities were reduced as pH_*e*_ increased, these activities were significantly higher than in medium at pH_*e*_ 7.4. These results seemed complementary to the transient increases in MMP expression (Fig. 4) and migration/invasion (Fig. 6).

### Adaptation to acidic pH_*e*_ is not simple selection of clones able to grow at pH_*e*_ 6.2

**Fig. 8:**
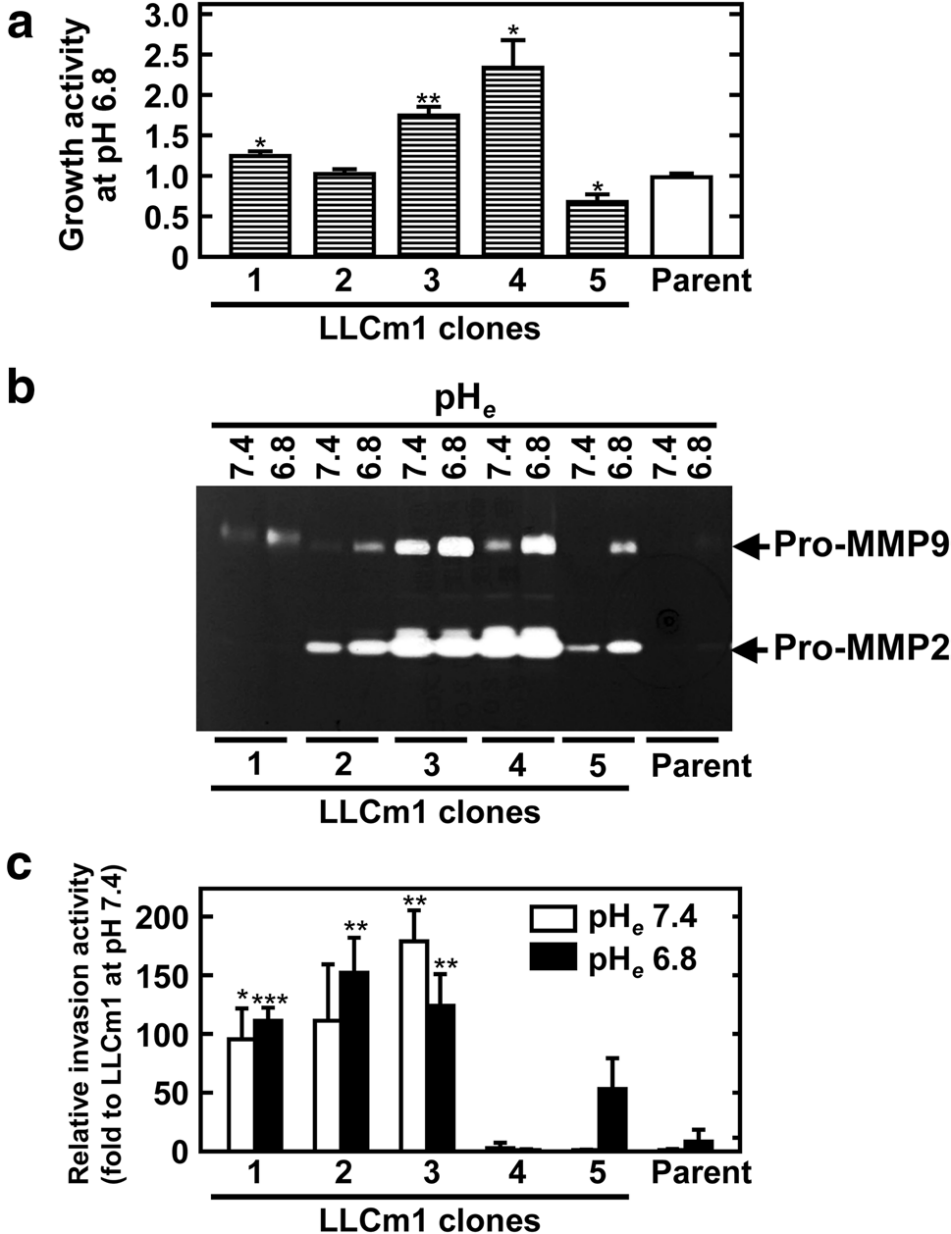
Growth at pH 6.8, MMP-2 and -9 secretion, and invasion properties of LLCm1 cell clones. LLCm1 cell clones in medium containing 10% FBS at pH 7.4 were subjected to limiting dilution. **a** Growth activity at pH 6.8. Cells were seeded at 8.5 × 10^4^ cells/cm^2^ in 24-well plates in pH 7.4 medium containing 10% FBS. Three hours later, the culture medium was changed to pH 6.8 medium containing 10% FBS and cells were further cultured for 24 h. Viable cell numbers were determined using the trypan blue dye exclusion method. Data expressed relative to the growth rate of parental LLCm1 cells. **b** Zymography. Cells pre-incubated with serum-free medium at pH 7.4 for 18 h were incubated in serum-free medium at pH 7.4 or 6.8 for an additional 24 h. MMPs in the CM were collected, concentrated by acetone precipitation, and analyzed by gelatin-zymography. **c** Invasion. Serum-starved cells were maintained in serum-free medium at pH 7.4 or 6.8. Culture medium was collected, and the cells were harvested by trypsinization and suspended in the same culture medium. Cells (5 × 10^5^) were placed onto Matrigel^®^-coated filters in transwell chambers. Cells that passed through onto the lower surface of the filter were counted after Giemsa staining. Representative results of two independent experiments are reported as mean ± SE (n = 3). *P < 0.05, **P < 0.01, ***P < 0.001

To test whether LLCm1A cells resulted from the simple clonal growth of preexisting acidic pH_*e*_ resistant cells rather than adaptation to acidic pH_*e*_, parental LLCm1 cells were cloned and their growth, MMP production and invasiveness were compared at pH_*e*_ 7.4 and pH_*e*_ 6.8 (Fig. 8). Of the LLCm1 cell clones assayed, clone 4 had the highest growth rate at acidic pH_*e*_. Although high amounts of MMP-2 and -9 were secreted, invasive activity was limited. These results suggested that the acquisition by LLCm1A cells of invasive activity was not simple clonal selection of preexisting acidic pH_*e*_-resistant cells but was also due to the dominant growth of “acidic pH_*e*_-adapted cells”. However, these findings also suggested the possibility of clonal growth of preexisting acidic pH_*e*_-resistant cells. Nevertheless, these results suggested that acidic pH_*e*_ shifted the heterogeneity of tumors to the accumulation of metastatic populations in the tumor microenvironment.

## Discussion

Metastatic activity has been associated with the tumor microenvironment, which consists of growth factors, the extracellular matrix, hypoxia, and acidic pH_*e*_. The acidic pH_*e*_ surrounding tumors is caused by the tumor cells’ secretion of lactic acid and CO_2_. Imaging technology has shown that tumors surrounded by pH_*e*_ are heterogeneous, consisting of acid donor and recipient cells [29]. This may be reflected in their relative use of MCT types, with donor cells mainly using MCT4 to secrete lactate/H^+^ [2] and recipient cells mainly using MCT1 to incorporate lactate/H^+^ [30]. Initially, we investigated the effect of transient acidic pH_*e*_ on metastatic phenotype [9, 26, 31, 32]. However, metastasis is thought to be caused by the dissemination of cells from the primary tumor, with tumor cells being affected by the tumor microenvironment including acidic pH_*e*_. This study therefore focused on the effects of adaptation to acidic pH_*e*_ especially on tumor invasion and metastasis. Transient acidification induces effective but reversible effects [9, 33], called the “memory effect” [33], which may be responsible for increased experimental metastasis induced by transient acidification [33, 34]. This study showed that tumor cell adaptation to acidic pH_*e*_ resulted in a metastatic phenotype. The high invasive activity of acidic pH_*e*_-adapted tumor cells was sustained through at least 28 serial passages (about 3 months) at neutral pH_*e*_, suggesting that the sustained invasive phenotype of these cells was likely not due to a memory effect but rather to an acquired phenotype. Thus, the acidic pH_*e*_-mediated acquisition of metastatic phenotype can likely be sustained in the circulation in vivo.

We also observed differences between cells exposed to transient acidification and those adapted to acidic pH_*e*_. Although *Krt5* mRNA expression was higher in acidic pH_*e*_-adapted LLCm1A than in LLCm1 cells, it was lower in the latter cells exposed to transient acidification. In contrast, *Trpm5* mRNA, which encodes a molecule involved in sensing acidic pH_*e*_ and whose overexpression in patients with melanoma and gastric cancer has been associated with shorter survival [26], was not affected by transient acidification. Although transient exposure of cells to acidic pH_*e*_-induced EMT [11, 12, 35], acidic pH_*e*_-adapted LLCm1A cells unexpectedly showed reduced expression of *Act2* mRNA, which encodes a mesenchymal marker, and increased expression of *Krt5* mRNA. Our working hypothesis was that cells of primary tumors affected for a long time by acidic microenvironments metastasize through the circulation. EMT is an important step, especially for dissemination of cells from primary tumors, whereas MET is involved in the establishment of secondary tumor formation [36]. This study assessed the in vivo metastatic potential of tumor cells injected through the tail vein, an experimental lung metastasis model evaluating steps in secondary tumor formation. Therefore, this experimental design reflected a situation in which primary tumor cells that had survived and adapted to acidic pH intravasate into the circulation, which is at pH_*e*_ 7.4. The acquired metastatic potential of acidic pH_*e*_-adapted tumor cells was sustained at physiological pH, with these cells playing an important role in secondary tumor formation through MET-like conversion.

Transient and chronic extracellular acidification have been reported to affect metabolic pathways through epigenetic alterations, including histone acetylation and DNA methylation [18, 37–39]. Adaptation or, in this study, resistance to acidic pH_*e*_ may also be regulated by these epigenetic alterations. Because highly proliferative cells consume glucose to generate ATP, and deoxyribose from the pentose-phosphate pathway, adaptation to extracellular acidification resulted in an escape from glucose dependence [37]. Cancer stem cells (CSC) and tumor initiating cells, which are resistant to drugs and divide asymmetrically, are thought to be the origin of tumor recurrence and metastasis [40]. CSCs are likely affected by, but are not responsible for, extracellular acidification [41], suggesting that cells adapted to acidic pH_*e*_ may have a partial CSC phenotype and may be a therapeutic target as much as CSCs [42].

The number of passages of cultured cells has been reported to affect tumor phenotype. Serial long-term or late passage was found to increase the metastatic activity of rat mammary adenocarcinomas [43], whereas serial passage of human pancreatic carcinomas had no effect on invasive activity [44]. Late passage was found to increase metastatic activity but not invasion through Matrigel^®^ [45], and late passage of human ovarian carcinoma cells increased MMP-9 but not MMP-2 expression [46]. Moreover, *KRT5* mRNA expression was higher in early than in late passage cells of the human mammalian epithelial MCF10A cell line, with late passage cells having a more mesenchymal phenotype than early passage cells [47], indicating that late passage decreased the stemness of human amnion mesenchymal cells [48]. In the present study, LLCm1A cells were derived from parental LLCm1 cells.

These parental cells were serially passaged in our laboratory and showed a stable phenotype, as assessed by morphology, MMP production, in vitro invasiveness and experimental metastasis. These activities were not increased by serial passage, in contrast to previous findings [12]. Moreover, tumor cell growth was extremely slow during adaptation to acid pH, but recovered after acidification, with adapted cells showing exponential growth without lag time just after seeding. Because a study of LLC cells found that the metastatic heterogeneity of tumors already pre-existed [49], we evaluated the heterogeneity of MMP production, invasiveness and growth potential at acidic pH_*e*_. Despite having growth potential at acidic pH_*e*_ with high MMP production, LLCm1 cell clone 4 did not have invasive activity, suggesting that the acquisition of invasive and metastatic ability is likely due not only to a simple effect of serial passage, but to adaptation to acidic pH_*e*_. Because our experiments could not completely distinguish between simple clonal selection and adaptation to acidic pH_*e*_, both remain possible. Our results showed, however, that acidic pH_*e*_ altered the tumor microenvironment, shifting tumor heterogeneity to the accumulation of a metastatic population. Because acidic pH_*e*_ was reported to induce the expression of sterol regulatory element-binding protein 2 (SREBP2) in pancreatic cancer cells [18], lipid homeostasis may regulate tumor metastasis in acidic microenvironments.

In conclusion, these findings suggest that prolonged tumor cell acidification induced a sustained invasive phenotype through a mechanism differing from that resulting from transient exposure to acidic pH_*e*_.

## Electronic supplementary material

Below is the link to the electronic supplementary material.

Supplementary material 1 (DOCX 15 kb). Table 1S: Primer sequences.

Supplementary material 2 (TIFF 215 kb). Figure 1S: pH growth dependence of LLCm1 and LLCm1A cells. LLCm1 cells (solid line) were inoculated at 4 × 10^5^ cells/cm^2^ per well in 24-well plates. After 1 day, the cells were cultured in medium at pH 7.4 (open circle), pH 6.8 (open square), or pH 6.2 (open diamond), each containing 10% FBS (arrow), and the medium was renewed on day 3. LLCm1A cells were cultured at a density of 1.5 × 10^5^ cells/cm^2^ in medium at pH 6.2 (filled diamond), with medium renewed on day 2. Representative results of two independent experiments are reported as mean ± SE. In some cases, error bars are hidden by the data symbol due to small values (n = 3)

Supplementary material 3 (TIFF 193 kb). Figure 2S: LLCm1A cells showed high potentials for MMP production and in vitro invasion regardless of neutralization. Cells were pre-incubated in the presence of 2% FBS at the desired pH, with no switch to physiological pH for 18 h. **A.** Cell morphology after culture for 9.5 h. Bar, 100 μm. **B.** Zymographic analysis (*Upper and Middle panels*). The intensity of the gelatin-lysed clear zone was determined using Image J software (National Institutes of Health, Bethesda, MD, USA). Data expressed as % of maximum (*Middle panel*, mean ± SE (n = 3)). In vitro cell invasiveness (*Lower panel*). Because cells incubated in serum-free medium at pH 6.2 became round in shape and detached rapidly from the culture dish, the cells were pre-incubated in medium containing 2% FBS for 18 h at each pH (although cell detachment could not be completely prevented as shown in panel A). Cells (5 × 10^5^) were harvested, resuspended in medium containing 20% FBS at each pH value and placed onto Matrigel^®^-coated filters in transwell chambers. Cells that passed through to the lower surface of the filter were counted after Giemsa staining. In some cases, error bars are hidden by the data symbol due to small values. Representative results of two independent experiments are reported as mean ± SE (n = 3). *P < 0.05, **P < 0.01, ***P < 0.001

## Data Availability

The datasets used and/or analyzed during the current study are available from the corresponding author on reasonable request.

## Abbreviations

CM: Conditioned medium
EMT: Epithelial-mesenchymal transition
FBS: Fetal bovine serum
HIF: Hypoxia inducible factor
LLC: Lewis lung carcinoma
MCT: Monocarboxylate transporter
MMP-9: Matrix metalloproteinase-9
PBS(-): Mg^2+^ and Ca^2+^-free phosphate-buffered saline
pH_*e*_: Extracellular pH
RT-qPCR: Reverse transcription-quantitative polymerase chain reaction
VEGF: Vascular endothelial cell growth factor
