# Supplementary material for: Adaptation to chronic acidic extracellular pH elicits a sustained increase in lung cancer cell invasion and metastasis
Source: Clin Exp Metastasis. 2019 Sep 5;37(1):133–44. doi: 10.1007/s10585-019-09990-1 (PMC7007909; doi:10.1007/s10585-019-09990-1)
Supplement: Supplementary file 1 — Supplementary material 1 (DOCX 15 kb). Table 1S: Primer sequences. [file 10585_2019_9990_MOESM1_ESM.docx]

**Supplemental Table 1. Primer sequences**

| Genes (Product) | Sequences | | Product (bp) |
| --- | --- | --- | --- |
| *Mmp2* (MMP-2, gelatinase A) | Forward: | 5'-agg cag tag agt aag ggg atc g-3' | 279 |
|  | Reverse: | 5'-tag aaa gtg ttc agg tat tgc act g-3' |  |
|  |  |  |  |
| *Mmp3* (MMP-3, stromelysin) | Forward: | 5'-tga agc att tgg gtt tct cta ct-3' | 134 |
|  | Reverse: | 5'-gat gcc ttc ctt gga tct ctt t-3' |  |
|  |  |  |  |
| *Mmp9* (MMP-9, gelatinase B) | Forward: | 5'-gcc ctg gaa ctc aca cga ca-3' | 85 |
|  | Reverse: | 5'-ttg gaa act cac acg cca gaa -3' |  |
|  |  |  |  |
| *Mmp13* (MMP-13, collagenase 2) | Forward: | 5'-tcc ctg gaa ttg gca aca aag-3' | 120 |
|  | Reverse: | 5'-gca tga ctc tca caa tgc gat tac-3' |  |
|  |  |  |  |
| *Mmp14* (MT-1MMP) | Forward: | 5'-tct tca agg agc gat ggt tct-3' | 182 |
|  | Reverse: | 5'-cag gga ggc ttc gtc aaa ca-3' |  |
|  |  |  |  |
| *Vim* (vimentin) | Forward: | 5'-gga cgt ttc caa gcc tga cct c-3' | 198 |
|  | Reverse: | 5'-ccg gta ctc gtt tga ctc ctg c-3' |  |
|  |  |  |  |
| *Cdh1* (E-cadherin) | Forward: | 5'-att gca agt tcc tgc cat cct c-3' | 145 |
|  | Reverse: | 5'-cac att gtc ccg ggt atc atc a-3' |  |
|  |  |  |  |
| *Cdh2* (N-cadherin) | Forward: | 5'-gtg acg act gaa cgg cag ga-3' | 121 |
|  | Reverse: | 5'-gca cgg tgc tag tgg act aca ga-3' |  |
|  |  |  |  |
| *Acta2* (α-smooth muscle actin) | Forward: | 5'-aga cgc tgc tcc agc tat gt-3' | 232 |
|  | Reverse: | 5'-cgg ata ctt cag cgt cag ga-3' |  |
|  |  |  |  |
| *keratin5* (keratin5) | Forward: | 5'-cat gaa cac caa gct ggc tc-3' | 141 |
|  | Reverse: | 5'-ccg tag cca gaa gag aca ctg-3' |  |
|  |  |  |  |
| *Snail* (snail) | Forward: | 5'-agg acg cgt gtg tgg agt tc-3' | 235 |
|  | Reverse: | 5'-tgg gag ctt ttg cca ctg tc-3' |  |
|  |  |  |  |
| *Slug* (slug) | Forward: | 5'-cat tcg aac cca cac att gcc-3' | 112 |
|  | Reverse: | 5'-aga gaa agg ctt ttc ccc agt g-3' |  |
|  |  |  |  |
| *Twist1* (twist1) | Forward: | 5'-gcc gga gac cta gat gtc att g-3' | 149 |
|  | Reverse: | 5'-acg ccc tga ttc ttg tga att tg-3' |  |
|  |  |  |  |
| *Twist2* (twist2) | Forward: | 5'-gca agc cag gac cca cc-3' | 100 |
|  | Reverse: | 5'-gtc atg agg agc cac aag gt-3' |  |
|  |  |  |  |
| *Zeb1* (zeb1) | Forward: | 5'-gct ggc aag aca acg tga aag-3' | 116 |
|  | Reverse: | 5'-agg ata aat gac ggc ggt gt-3' |  |
|  |  |  |  |
| *Zeb2* (zeb2) | Forward: | 5'-aga ctt cac aga tcg agc ct-3' | 146 |
|  | Reverse: | 5'-cct cct ggg att ggc ttg tt-3' |  |
|  |  |  |  |
| *Actb* (β-actin) | Forward: | 5'-cat ccg taa aga cct cta tgc caa c-3' | 85 |
|  | Reverse: | 5'-atg gag cca ccg atc cac a-3' |  |
