# Supplementary figures and images for: Adaptation to chronic acidic extracellular pH elicits a sustained increase in lung cancer cell invasion and metastasis
Source: Clin Exp Metastasis. 2019 Sep 5;37(1):133–44. doi: 10.1007/s10585-019-09990-1 (PMC7007909; doi:10.1007/s10585-019-09990-1)

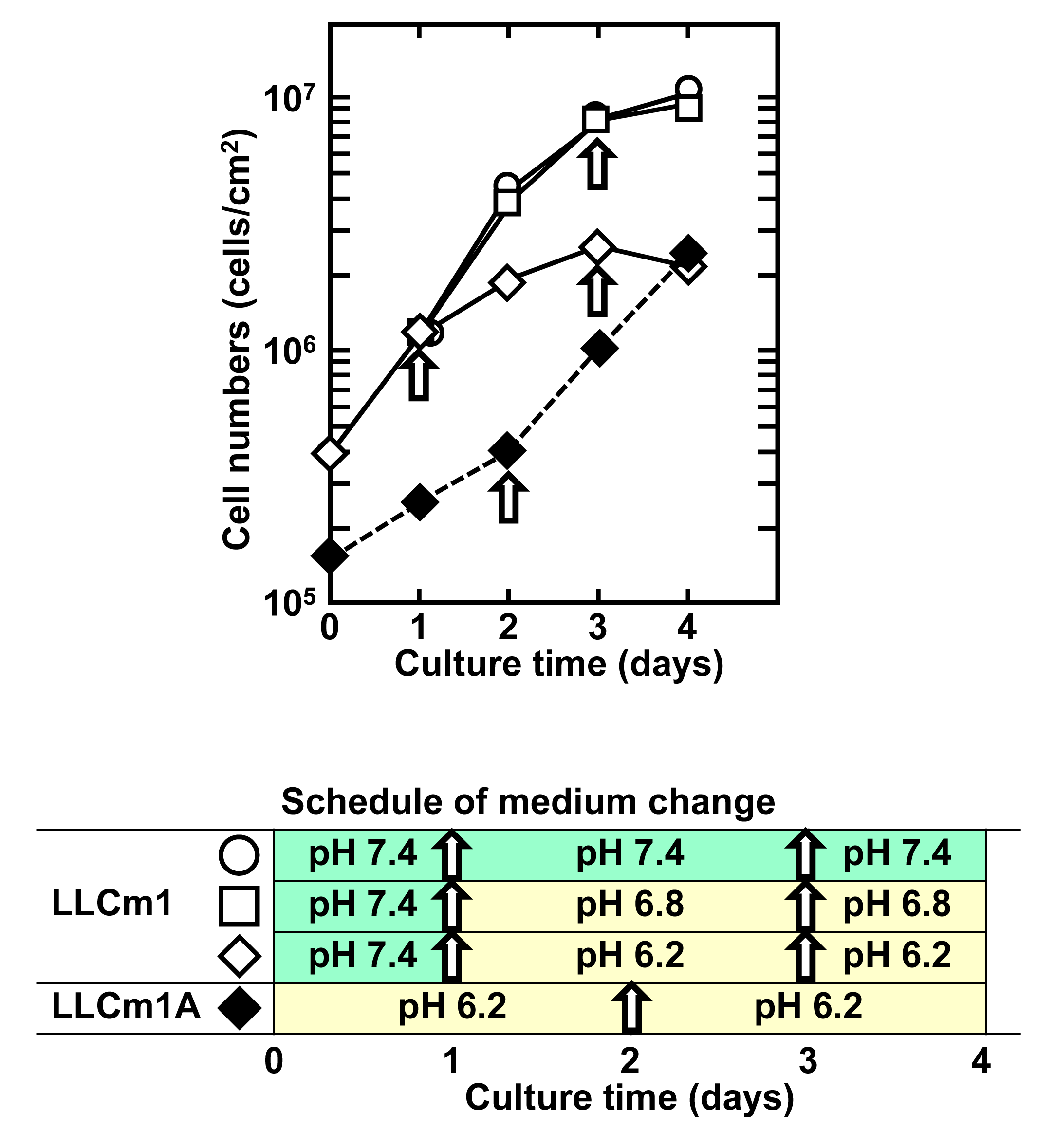

Supplement: Supplementary file 2 — Supplementary material 2 (TIFF 215 kb). Figure 1S: pH growth dependence of LLCm1 and LLCm1A cells. LLCm1 cells (solid line) were inoculated at 4 × 10^5^ cells/cm2 per well in 24-well plates. After 1 day, the cells were cultured in medium at pH 7.4 (open circle), pH 6.8 (open square), or pH 6.2 (open diamond), each containing 10% FBS (arrow), and the medium was renewed on day 3. LLCm1A cells were cultured at a density of 1.5 × 10^5^ cells/cm2 in medium at pH 6.2 (filled diamond), with medium renewed on day 2. Representative results of two independent experiments are reported as mean ± SE. In some cases, error bars are hidden by the data symbol due to small values (n = 3) [file 10585_2019_9990_MOESM2_ESM.tif]

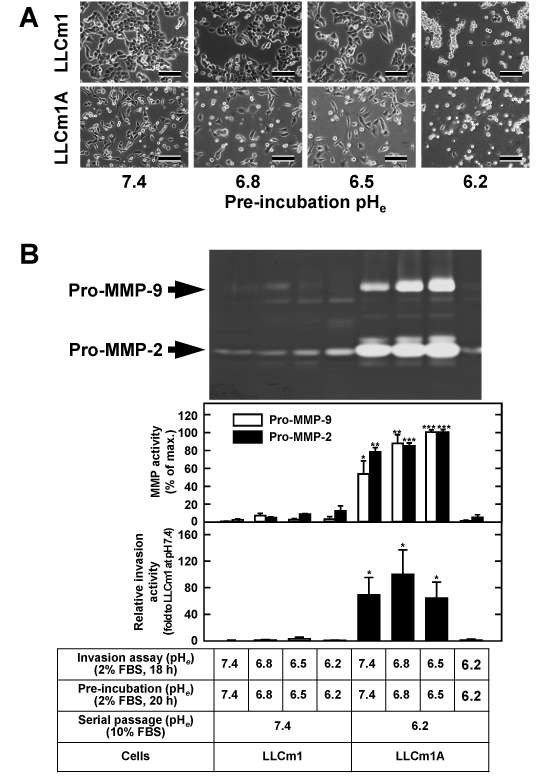

Supplement: Supplementary file 3 — Supplementary material 3 (TIFF 193 kb). Figure 2S: LLCm1A cells showed high potentials for MMP production and in vitro invasion regardless of neutralization. Cells were pre-incubated in the presence of 2% FBS at the desired pH, with no switch to physiological pH for 18 h. A. Cell morphology after culture for 9.5 h. Bar, 100 μm. B. Zymographic analysis (Upper and Middle panels). The intensity of the gelatin-lysed clear zone was determined using Image J software (National Institutes of Health, Bethesda, MD, USA). Data expressed as % of maximum (Middle panel, mean ± SE (n = 3)). In vitro cell invasiveness (Lower panel). Because cells incubated in serum-free medium at pH 6.2 became round in shape and detached rapidly from the culture dish, the cells were pre-incubated in medium containing 2% FBS for 18 h at each pH (although cell detachment could not be completely prevented as shown in panel A). Cells (5 × 10^5^) were harvested, resuspended in medium containing 20% FBS at each pH value and placed onto Matrigel®-coated filters in transwell chambers. Cells that passed through to the lower surface of the filter were counted after Giemsa staining. In some cases, error bars are hidden by the data symbol due to small values. Representative results of two independent experiments are reported as mean ± SE (n = 3). *P < 0.05, **P < 0.01, ***P < 0.001 [file 10585_2019_9990_MOESM3_ESM.tif]
